# Modeling the Interaction of Coronavirus Membrane Phospholipids with Photocatalitically Active Titanium Dioxide

**DOI:** 10.1021/acs.jpclett.3c01372

**Published:** 2023-06-21

**Authors:** Iván Soriano-Díaz, Eros Radicchi, Beatrice Bizzarri, Olivia Bizzarri, Edoardo Mosconi, Muhammad Waqar Ashraf, Filippo De Angelis, Francesca Nunzi

**Affiliations:** † Department of Chemistry, Biology and Biotechnology, University of Perugia, Via Elce di Sotto 8, 06123 Perugia, Italy; ‡ Nanomaterials Research Group, Department of Biotechnology, University of Verona, Strada Le Grazie 15, 37134 Verona, Italy; § Computational Laboratory for Hybrid/Organic Photovoltaics (CLHYO), Consiglio Nazionale delle Ricerche (CNR) - Istituto di Scienze e Tecnologie Chimiche “Giulio Natta” - SCITEC, Via Elce di Sotto 8, 06123 Perugia, Italy; ∥ Department of Natural Sciences and Mathematics, College of Sciences and Human Studies, Prince Mohammad Bin Fahd University, Khobar, Dhahran 34754 Saudi Arabia; ⊥ SKKU Institute of Energy Science and Technology (SIEST), Sungkyunkwan University, Suwon 440-746, Korea; ○ Instituto de Ciencia Molecular, Universidad de Valencia, 46980 Paterna, Spain

## Abstract

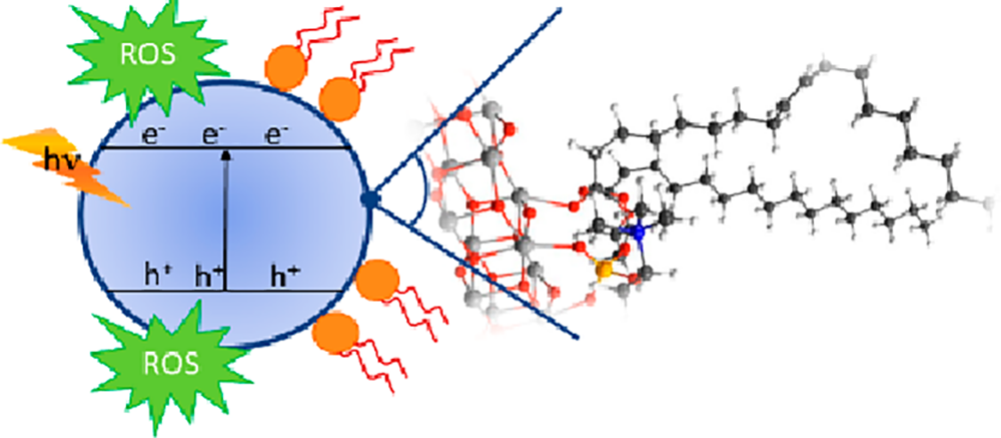

The outbreak of viral infectious diseases urges airborne droplet and surface disinfection strategies, which may rely on photocatalytic semiconductors. A lipid bilayer membrane generally encloses coronaviruses and promotes the anchoring on the semiconductor surface, where, upon photon absorption, electron–hole pairs are produced, which can react with adsorbed oxygen-containing species and lead to the formation of reactive oxygen species (ROSs). The photogenerated ROSs may support the disruptive oxidation of the lipidic membrane and pathogen death. Density functional theory calculations are employed to investigate adsorption modes, energetics, and electronic structure of a reference phospholipid on anatase TiO_2_ nanoparticles. The phospholipid covalently bound on TiO_2_, engaging a stronger adsorption on the (101) than on the (001) surface. The energetically most stable structure involves the formation of four covalent bonds through phosphate and carbonyl oxygen atoms. The adsorbates show a reduction of the band gap compared with standalone TiO_2_, suggesting a significant interfacial coupling.

Despite the enormous progress of modern medicine, different pathogenic microorganisms still threaten human health, causing not only dangerous infectious diseases but also consistent economic losses. Effective and environmentally friendly strategies to accomplish the disinfection of broad-spectrum pathogens are urgently needed to mitigate the transmission and reduce the consequences on human society.

The outbreak of Coronavirus Disease 2019 (COVID-19), caused by Severe Acute Respiratory Syndrome Coronavirus 2 (SARS-CoV-2),^1,2^ points out that the transmission spreads through direct or indirect contact via virus-containing airborne droplets or contaminated surfaces of objects.^3−7^ Understanding contamination via aerosols and surfaces is crucial to plan effective preventive measures and disrupt disease transmission via environmental routes.^8,9^ An optimal strategy should involve the employment of air-purifying systems and antiviral surfaces next to the commonly used high hygiene standards, personal protective equipment, and vaccination programs. Antiviral coatings for surfaces that are handled daily may kill the viruses and avoid transmission through the respiratory droplets deposited on the surfaces. Semiconductor nanomaterials, widely employed in environmental remediation applications,^10−12^ can be efficiently employed in the development of air-purifying systems and universal antiviral protection coatings.^13,14^ Semiconductor nanoparticles (NPs) have a high surface area, thus involving a higher number of reactive centers on the surface that can promote the chance for a higher efficiency in the photocatalytic processes. The most widely used material is titanium dioxide (TiO_2_),^15^ that has proven to be efficient in photodegradation of organic pollutants, self-cleaning properties, and photoinduced bacterial and virus disinfection.^16−18^ Among the various TiO_2_ polymorph phases, anatase is the more stable form for NPs with diameter below ∼20 nm, while rutile is the most thermodynamically stable bulk phase.^19^ Anatase TiO_2_ NPs usually show a higher photocatalytic activity under ultraviolet light in comparison to rutile, and its origin is probably related to multiple factors, such as morphology, size, defect chemistry, and/or adsorbates.^17,19^ The (101) facet is the most stable facet in anatase TiO_2_, but NPs with a larger percentage of the more reactive (001) facet can be synthesized by adjusting reaction parameters.^20,21^

Upon photon absorption by TiO_2_ NPs, electron–hole pairs are produced that subsequently migrate to the surface. Here, holes can react with adsorbed H_2_O or OH^–^ to produce highly reactive hydroxyl radicals (·OH), while electrons can react with O_2_ to produce superoxide radical ions (·O_2_^–^), which are further reduced to OH. Since these radicals are highly reactive, they are referred to as reactive oxygen species (ROSs). The photocatalytically produced charge carriers and ROSs can oxidize organic compounds adsorbed on the TiO_2_ surface, including those constituting the outer virus membrane, thus promoting the death of the pathogen. The exact mechanism by which TiO_2_ NPs reduce the infectivity of the viruses is still debated. While each pathogen has its unique characteristics, contributing to pathology and host response, some common features can be outlined in viral inactivation pathways. Most viral respiratory infections are caused by RNA viruses. Among these, coronaviruses are enveloped viruses, where RNA is packaged within an outer lipid bilayer membrane, derived from the host cell membrane. The SARS-CoV-2 fatty membrane contains virus proteins and acts like a bag holding and protecting viral RNA. The membrane needs to be sufficiently stable to protect RNA from the surrounding environment but not so stable that it cannot break open inside the host cell to release the RNA. This balance between structural stability and the ability to release RNA is essential for the transmission and the replication of the virus but also renders the membrane susceptible to be destroyed upon interaction with the photoinduced ROSs and charge carriers on the TiO_2_ surface. The killing mechanism is likely more efficient when close contact between the virus and the TiO_2_ surface, where the ROSs are generated, is engaged. The anchoring of the virus on the titania surface can be accomplished by the formation of covalent bonds between undercoordinated titanium atoms and nucleophilic sites on the outer part of the viral membrane, mainly constituted by bilayer phospholipids. Many studies have investigated the adsorption/interaction of various phospholipids derivates on the titanium dioxide surface,^22−30^ suggesting that their polar head groups play a major role in the binding with the TiO_2_ surface.^22−24,28,29^ Fundamental studies have targeted the nature of the interaction of various phosphonic acid derivates on the TiO_2_ surface, pointing out how phosphate derivates may strongly bind to oxide surfaces via Ti–O–P bonds, formed between the phosphoryl oxygen and undercoordinated Ti^4+^ sites.^30−33^ Theoretical investigations addressed a mono-, bi-, or tridentate bonding mode for the phosphonic acid derivates on different materials, with the bidentate binding mode involving both a phosphate group bridging between two Ti^4+^ sites or chelating to a single Ti^4+^ site. In particular, previous density functional theory (DFT) studies on the adsorption of phosphonic acid on TiO_2_ cluster models found that the monodentate mode through the coordination of the P=O group is slightly favored over the bidentate modes.^34−36^ Employing periodic localized basis set calculations with the B3LYP functional, Bermudez predicted a bidentate adsorption mode for the dimethysphosponate on the rutile surface.^37^ Using periodic DFT calculations in combination with GIPAW NMR calculations and experimental IR and solid-state ^17^O and ^31^P NMR spectroscopies, Tielens et al. found that the bidentate geometry is thermodynamically favored on the (001) and (101) hydrated anatase TiO_2_ surface.^38^ The same conclusion was previously asserted by Luschtinetz et al. by tight binding DFT calculations.^39^ Di Valentin et al. investigated the adsorption of an n-butylphosphonic acid on the anatase (101) TiO_2_ surface by DFT calculations, pointing out that the bonding mode also depends on the coverage of the acid on the oxide surface.^40^

In this Letter, we provide a systematic study of the adsorption modes and energetics of a reference phospholipid, as a model for the real bilayer membrane, on anatase TiO_2_ NP surface by means of DFT calculations. Among the various phospholipids, 1-palmitoyl-2-oleoyl-sn-glycero-3-phosphocholine (POPC) has been selected, being typically considered one of the model lipids for biophysical experiments.^41,42^ POPC is constituted by a hydrophilic head (phosphate group, choline residue, and oxygen atoms derived by the glycerol) and a hydrophobic tail (two different fatty acids, palmitate and oleate). Despite previous fundamental studies describing the anchoring of phosphate derivatives on the titanium oxide surface, the POPC phospholipid case can be rather different, because of the peculiar structure of its skeleton, characterized by four nucleophile sites, i.e., two phosphoryl oxygen sites and two ester carbonyl oxygen sites, that are potential anchoring sites on the oxide surface, and by two long acyl chains, giving rise to possible dispersion interactions and therefore affecting the overall adsorption strength. TiO_2_ NPs exposing both the (101) and (001) surfaces have been considered and modeled by extended models, which are reproducing the structural and electronic properties of the real TiO_2_ NPs. Once the virus is anchored on the TiO_2_ surface by means of Ti–O bonds between the POPC nucleophile oxygen sites and the electrophile Ti sites, the oxidative reaction on the POPC double C–C bond can eventually take place by the intervention of the ROSs, thus leading to the inhibition of the virus pathogen effect.

To systematically examine the various feasible POPC adsorption configurations on the TiO_2_ NP surface at a reasonable calculation time, a simplified model of the POPC phospholipid was employed, where the two aliphatic chains were replaced with methyl groups, hereafter labeled as PC (see Figure 1). Afterward, the absorption of the real POPC phospholipid on the oxide surface was considered, limited to the more stable energy configurations.

**Figure 1:**
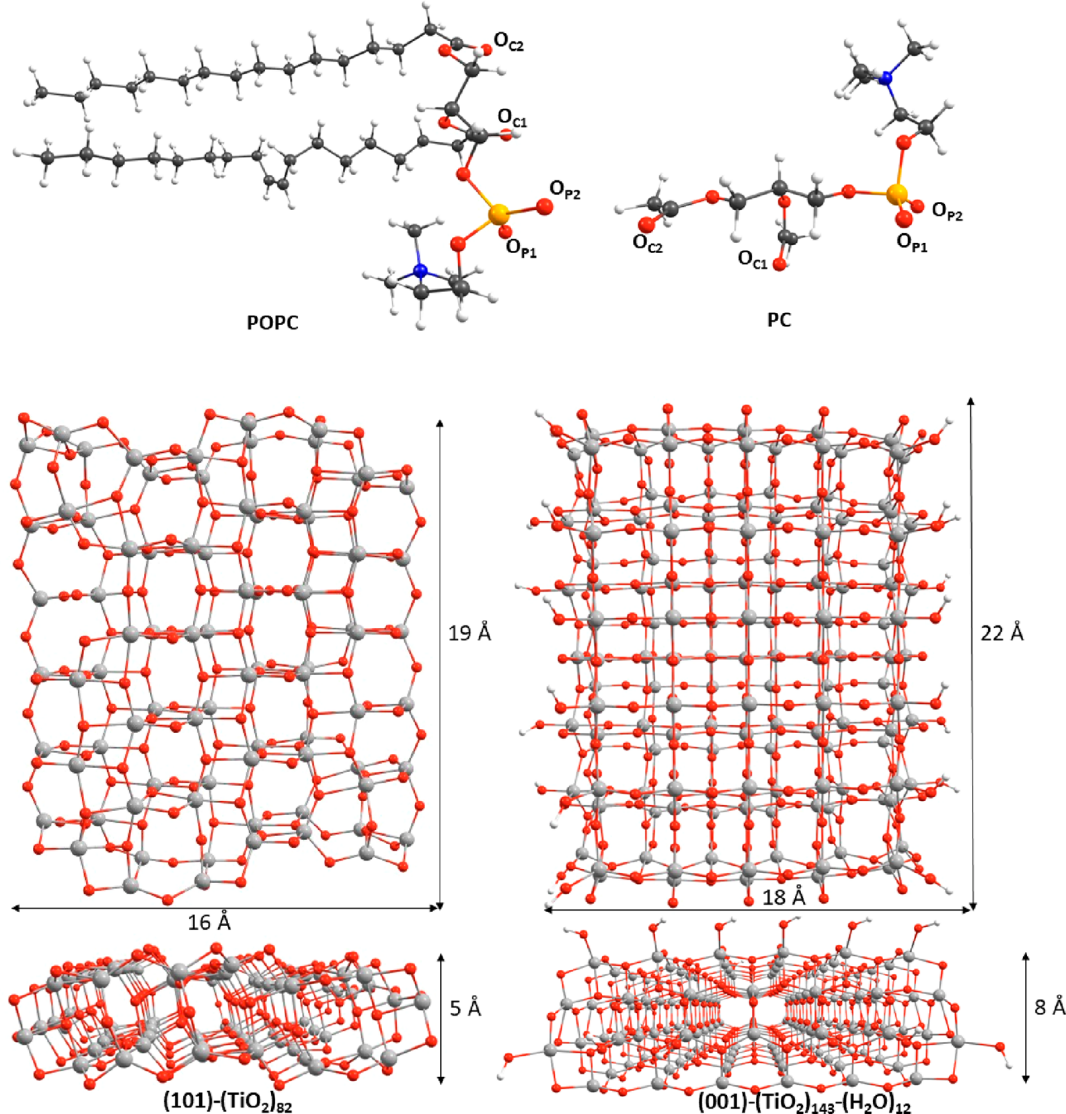
Optimized geometries for the POPC (top left) and PC (top right) lipid model, together with the labeling of the oxygen nucleophilic sites, the (TiO_2_)_82_ cluster model, exposing the (101) surface (bottom left), and the (TiO_2_)_143_-(H_2_O)_12_ cluster model, exposing the (001) surface (bottom right, top and side views). Carbon atoms in dark gray, titanium in light gray, oxygen in red, phosphorus in orange, nitrogen in blue, and hydrogen in white.

According to the cluster approach for the description of TiO_2_ based on the original concept of Persson and co-workers,^43^ we considered three reduced cluster models to reproduce the anatase TiO_2_ NP features,^44^ as reported in Figures 1 and S1. The smaller cluster model is constituted by 38 TiO_2_ units, (TiO_2_)_38_ (Figure S1), and it has been employed to quickly verify the adsorption site possibilities of the reduced POPC model on the oxide surface (details are presented in the Supporting Information). Remarkably, this cluster has been extensively characterized in previous studies^34,43,45−48^ and has shown to have electronic and optical properties comparable to those of TiO_2_ NPs a few nanometers in size.^49,50^ Afterward, we considered two more extended clusters, (TiO_2_)_82_ and (TiO_2_)_143_-(H_2_O)_12_, reproducing the (101) and (001) anatase TiO_2_ surfaces, respectively (see Figure 1). The (101)-terminated (TiO_2_)_82_ model is an almost square TiO_2_(101) two-layer anatase slab ca. 2 nm long, with three rows of five- (Ti_5c_) and six-coordinated (Ti_6c_) surface Ti sites, and it has been computed to have a density of states comparable to that of the corresponding periodic surface model.^51,52^ Due to the different topology of the (001) vs (101) surface, by cutting a periodic (001)-surface slab ca. 2 nm long, a nonstoichiometric (Ti_*n*_O_2*n*–x_) cluster was gained, missing *x* oxygen atoms and with a positive charge of +2*x*. Therefore, a number of OH^–^ groups equal to 2*x* were added to the cluster to compensate the global charge and saturate the tetracoordinated Ti (Ti_4c_) atoms, setting up a three-layer (001)-terminated anatase slab with global stoichiometry (TiO_2_)_143_-(H_2_O)_12_. The electronic and optical properties of the (101)-(TiO_2_)_82_ and (001)-(TiO_2_)_143_-(H_2_O)_12_ cluster models have been considered in detail in refs (45, 51, and 52). It is worth noting that these two cluster models expose roughly the same active surface area but differ in the number of surface layers because of the peculiar saturation scheme adopted for the (001) model. Nevertheless, they are large enough to avoid possible spurious POPC–oxide interactions at the cluster border, related to the finite cluster size. Since the present investigation is focused on the POPC adsorption modes, the employed stoichiometric TiO_2_ NPs models, not including potential structural defects, such as oxygen vacancies or adsorbed water molecules, return an adequate description of the adsorbates while reducing the complexity of the calculations.

Electronic structure calculations and geometry optimizations were carried at the DFT PBE level of theory^53^ with nonlocal van der Waals interactions included through the rVV10 scheme.^54^ We employ the freely available CP2K suite of codes,^55−57^ together with the Quickstep module,^57^ which allows efficient and fast DFT calculations on extended systems. Double-ζ polarized basis sets are adopted for the wave functions,^58^ while Goedecker–Teter–Hutter pseudopotentials^59,60^ are used to account for core–valence interactions. We consider a cutoff of 400 Ry for the expansion of the electron density in plane waves. Periodic boundary conditions (PBC) were set in order to treat each system independently as a particle by increasing cell parameters at least 10 Å in each spatial direction, to make sure that no interactions occur between the clusters.

We first optimized the geometries of the bare PC, POPC, and TiO_2_ models, followed by the optimization of the corresponding POPC-adsorbed structures in various configurations. The geometry optimizations have been carried out both with and without the inclusion of the dispersion forces, finding negligible differences in the structural parameters, while the adsorption energies are found to be highly affected by the inclusion of the van der Waals contribution (see Tables S1 and S2). In the following, the discussion is limited to the geometrical and energy values computed with the inclusion of the dispersion forces.

The adsorption energy of the phospholipid on the TiO_2_ surface is defined as
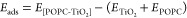
where *E*_[POPC-TiO_2_]_ is the total energy of the POPC-TiO_2_ (or PC-TiO_2_) whole system, *E*_TiO_2__ is the energy of the TiO_2_ NP, and *E*_POPC_ is the energy of the POPC (or PC) molecule in the gas phase.

Physisorption, as well as chemisorption, with the formation of up to four Ti–O linkages, are considered. The physisorption structures are labeled as P1, while, concerning the chemisorption adsorbates, the Ti–O–P monodentate structures are labeled as M1 and the Ti–O–P bidentate structures are labeled as B1, B2, and B3. The potential Ti–O–C bonds, involving the ester carbonyl oxygen sites or the acyl chain dispersion interactions, further stabilizing the adsorbate, are not explicitly considered in the label structure. The results are reported in Figures 2–5 and Table 1.

**Table 1:** Main Bond Distances (in Angstroms), Adsorption Energies (*E*_ads_), and HOMO–LUMO Energy Gap (Δ_H–L_, in electronvolts) for the PC/POPC Adsorbates on the (101)-(TiO_2_)_82_ (top) and (001)-(TiO_2_)_143_-(H_2_O)_12_ (bottom) NPs

**(101)-(TiO**_**2**_**)**_**82**_						
	**PC-P1**	**PC-M1**	**PC-B1**	**PC-B2**	**POPC-B2**	**POPC-B3**
**Ti-O**_**1P**_	3.68	1.98	2.15	2.19	2.14	2.15
**Ti-O**_**2P**_	5.14	4.04	2.17	2.13	2.15	2.18
**Ti-O**_**3C**_	6.03	5.27	4.09	2.37	2.17	2.36
**Ti-O**_**4C**_	3.45	3.30	5.03	2.27	2.29	2.18
**P-O**_**1P**_	1.48	1.55	1.51	1.52	1.51	1.51
**P-O**_**2P**_	1.50	1.47	1.51	1.51	1.52	1.52
**C-O**_**3C**_	1.22	1.21	1.21	1.23	1.22	1.23
**C-O**_**4C**_	1.22	1.23	1.21	1.22	1.23	1.22
**E**_**ads**_	–0.91	–2.43	–2.04	–3.46	–4.08	–5.59
**Δ**_**H-L**_	0.57	1.56	2.05	2.16	1.41	1.11

**(001)-(TiO**_**2**_**)**_**143**_**-(H**_**2**_**O)**_**12**_						
	**PC-P1**	**PC-M1**	**PC-B1**	**PC-B2**	**POPC-B2**	**POPC-B3**
**Ti-O**_**1P**_	4.84	2.10	2.13	2.11	2.13	2.11
**Ti-O**_**2P**_	6.03	3.38	2.25	2.12	2.08	2.15
**Ti-O**_**3C**_	5.75	5.42	3.67	2.30	2.29	2.28
**Ti-O**_**4C**_	2.70	4.46	2.99	2.97	3.03	2.97
**P-O**_**1P**_	1.49	1.53	1.53	1.51	1.51	1.50
**P-O**_**2P**_	1.50	1.49	1.53	1.52	1.52	1.51
**C-O**_**3C**_	1.22	1.22	1.22	1.23	1.24	1.23
**C-O**_**4C**_	1.23	1.22	1.22	1.22	1.22	1.22
**E**_**ads**_	–0.72	–1.72	–2.22	–2.96	–3.21	–4.53
**Δ**_**H-L**_	0.31	2.18	1.98	2.22	1.41	1.93

**Figure 2:**
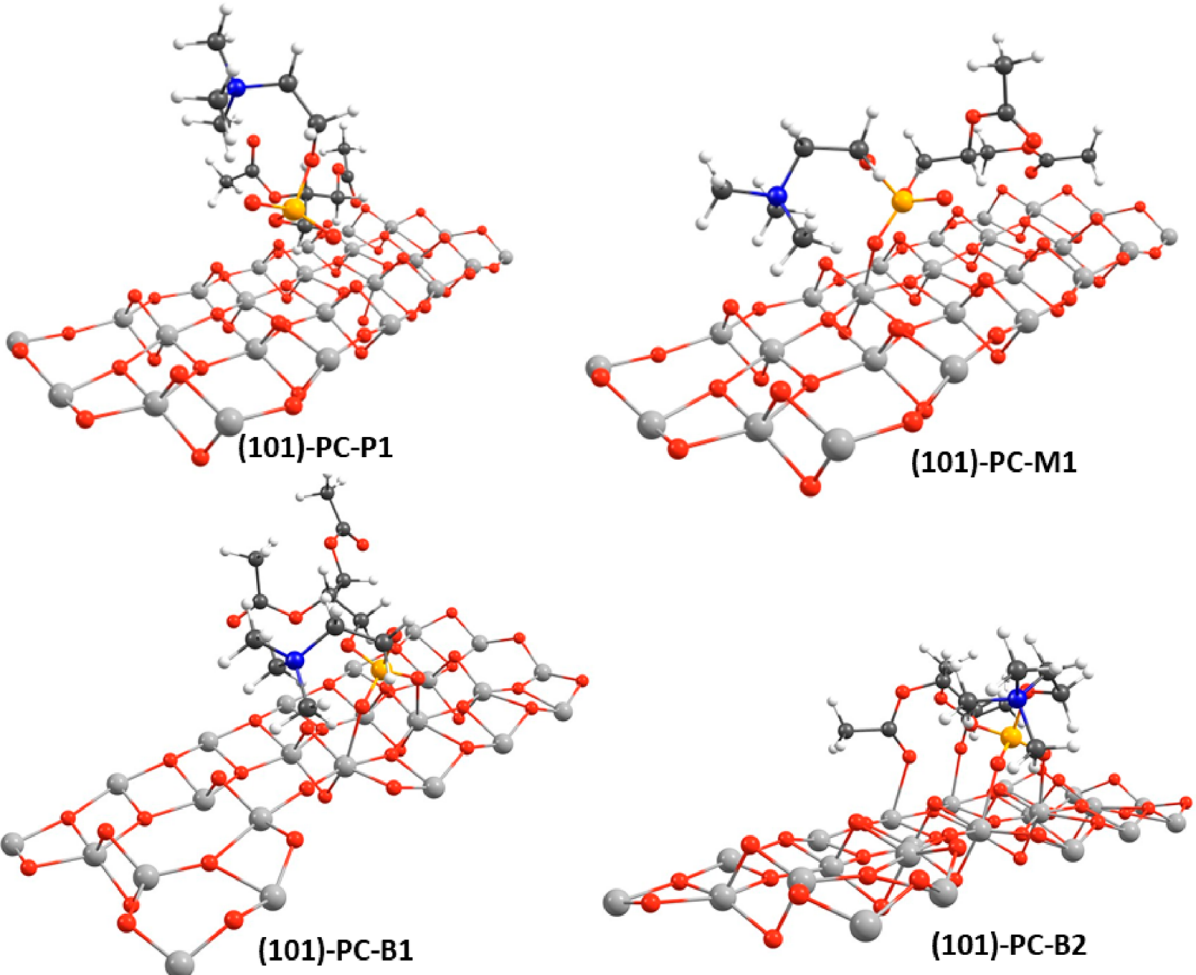
Optimized geometries for the adsorption of PC on the (101)-(TiO_2_)_82_ cluster (only a limited surface section is shown for clarity); see text for structure labeling. Titanium atoms in light gray, oxygen in red, carbon in dark gray, phosphorus in orange, nitrogen in blue, and hydrogen in white.

**Figure 3:**
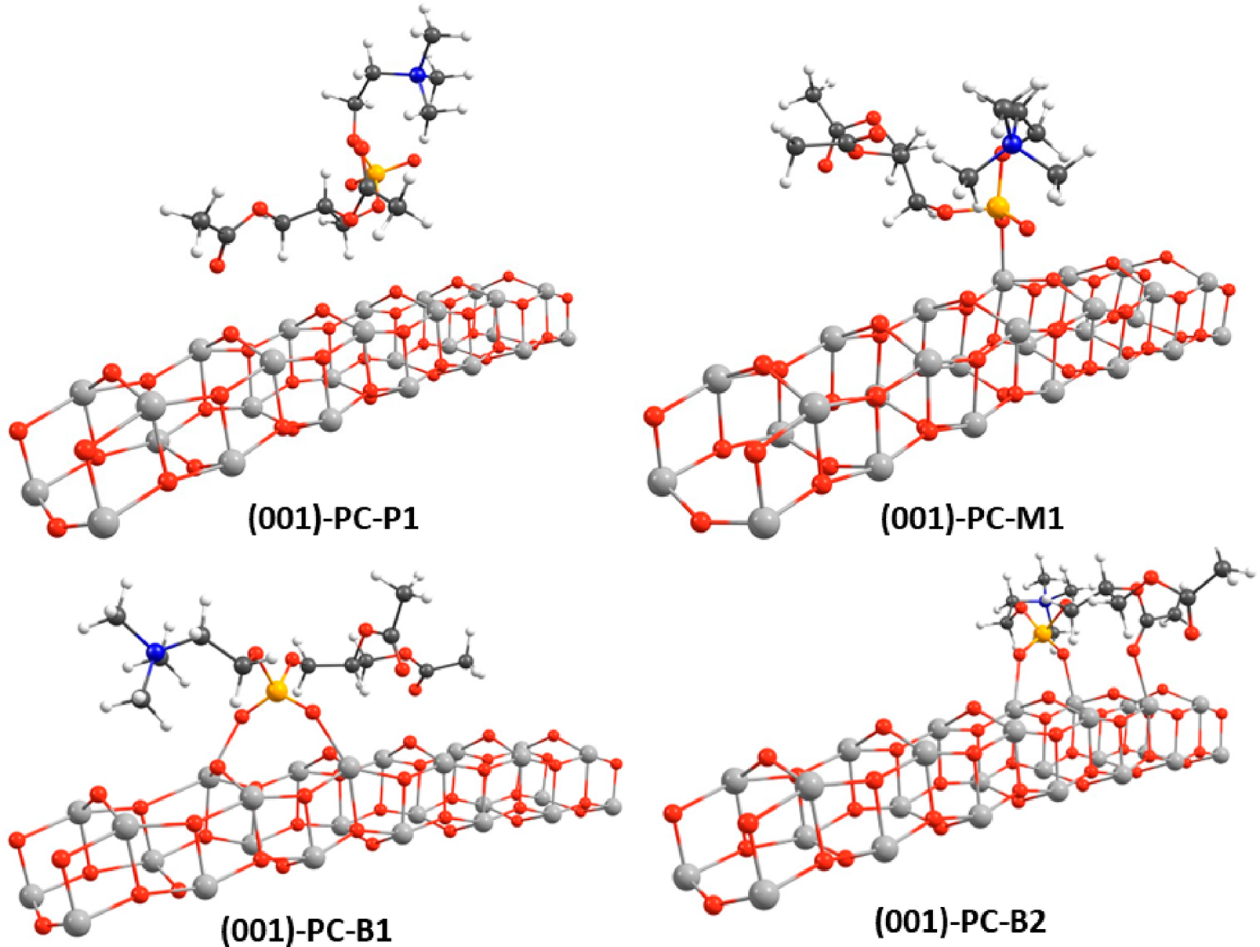
Optimized geometries for the adsorption of PC on the (001)-(TiO_2_)_243_-(H_2_O)_12_ cluster (only a limited surface section is shown for clarity); see text for structure labeling. Titanium atoms in light gray, oxygen in red, carbon in dark gray, phosphorus in orange, nitrogen in blue, and hydrogen in white.

*(101)-(TiO_2_)_82_-PC and (001)-(TiO_2_)_143_-(H_2_O)_12_-PC Structures*. The (101)-PC-P1 and (001)-PC-P1 structures consider physisorption of the PC phospholipid on the titanium oxide surface, the Ti-O distances being between 2.70 and 6.03 Å, and they have adsorption energies of −0.91 and −0.71 eV, respectively. The PC residue shows almost the same orientation as in the (TiO_2_)_38_ cluster model, with the phosphate residue lying close to the surface and the choline residue lying far from it.

The (101)-PC-M1 and (001)-PC-M1 structures consist of a monodentate binding mode of one phosphate oxygen on the Ti_5c_ site of the oxide surface, with a Ti–O bond distance of 1.98 and 2.10 Å, respectively, while the adjacent P–O bond lengthens to 1.55 and 1.53 Å, respectively, being 1.49 Å in the free PC molecule. Adsorption energies of −2.43 and −1.72 eV have been computed for the (101)-PC-M1 and (001)-PC-M1 structures, respectively. It is interesting to compare these values with those obtained by Soria et al. for the monodentate adsorption of dihydrogen phosphate (H_2_PO_4_^–^) on the TiO_2_ (101) surface at a B3LYP level of theory.^61^ They found a Ti–O bond distance of 1.92 Å and an adsorption energy of only −0.86 eV. Despite possible differences due to the employed computational approach, we can argue that the choline and the diglycerol ester residues play a major role in the stabilization of the PC adsorbate on the (101) surface.

The (101)-B1 and (001)-B1 structures show a bidentate binding mode, where the two oxygens of the polyphosphate group are anchored to the Ti_5c_ atoms of the oxide surface, with the Ti–O bond distances equal to 2.15/2.17 and 2.13/2.25 Å, respectively, and the adjacent P–O bonds lengthen to 1.51 and 1.53 Å, respectively. The adsorption energies are computed to be −2.04 and −2.22 eV for the (101)-B1 and (001)-B1 structures, respectively. The bidentate H_2_PO_4_^–^ adsorbates in ref (61) show an additional feature with respect to the present PC adsorbates, which is the presence of a hydrogen bond between a phosphate hydrogen and a O_2c_ site, so that short Ti–O bond distances (1.90–2.02 Å) and significant adsorption energies (−1.66 and −2.01 eV) are computed for the adsorbates, thus suggesting an almost comparable interaction strength with respect to the PC moiety. The (101)-B1 structure is 0.39 eV higher in energy than the (101)-M1 structure, thus suggesting that on the (101) NP surface the monodentate binding mode is energetically favored, while the reverse is found for the (001) NP surface, the (001)-B1 structure being more stable in energy than the (001)-M1 structure by 0.50 eV.

Finally, the (101)-B2 and (001)-B2 structures describe a bidentate binding mode with respect to the phosphate oxygens, with bond distances of 2.13/2.19 and 2.11/2.12 Å, respectively. In addition, the (101)-B2 adsorbate shows two covalent Ti–O–C bonds (2.27/2.37 Å), involving the ester carbonyl oxygen sites, while the (001)-B2 adsorbate engages only one Ti–O–C bond (2.30 Å). In both adsorbates, the adjacent P–O bonds lengthen from 1.49 Å in the free PC to 1.51 and 1.52 Å, while the adjacent C–O bonds keep the same distance as in the free PC (1.22 Å). These data suggest a stronger interaction of acid titanium sites with the phosphoryl oxygen sites with respect to the ester carbonyl oxygen sites. The making of the additional covalent Ti–O bonds leads to the most energetically stable structure for each (101) and (001) TiO_2_ cluster, with computed values of even −3.46 and −2.96 eV for the (101)-B2 and (001)-B2 structures, respectively. Interestingly, a stabilization energy of even 1.42/0.74 eV is attained passing from the (101)-B1/(001)-B1 adsorbates to (101)-B2/(001)-B2, where covalent bonds with the ester carbonyl oxygen atoms are engaged.

The electronic structure for PC adsorbed on the (101) and (001) TiO_2_ anatase clusters has been analyzed (see Figure 4 and Table 1). Even though it is well-known that the GGA-DFT approach strongly underestimates the TiO_2_ HOMO–LUMO energy gap,^44^ it is interesting to verify if the various adsorption modes of the PC on the TiO_2_ NP surface affect the energy gap of the pristine TiO_2_ NP, determining a variation of its value and/or the formation of new states inside the material gap. These features are particularly appealing for the TiO_2_ photocatalytic properties, since they would involve the extension of the adsorption edge in the visible spectrum. For the bidentate B2 adsorption mode, for which the strongest adsorption energies have been found among the (101)-TiO_2_-PC and (001)-TiO_2_-PC structures, the energy gap decreases only by 0.20/0.02 eV with respect to the pristine (101)/(001) TiO_2_ NP. Moreover, the DOS reported in Figure 4 shows that the PC molecular states lie in the same energy range of the valence band, mainly constituted by O (TiO_2_) atoms, and higher in energy with respect to the lower part of the conduction band, mainly constituted by Ti atoms, so that the adsorbate energy gap results completely empty. This feature suggests a strong electronic coupling of the PC molecular orbitals with the titania bands, which is important for an efficient electron transfer between the titania oxide and the biomolecule. As clearly shown from Figure 4, the other adsorbate configurations, having lower adsorption energies, have a peculiar DOS, where new states appear in the energy gap, constituted by PC molecular states, with the contribution from the PC oxygen atoms being prevalent. In particular, the physiosorbed P1 configurations, where the interaction lacks any chemical contribution, show a closure of the energy gap to a value of only 0.57/0.31 eV for the (101)/(001)-TiO_2_ adsorbate, since the occupied frontier PC orbitals, mainly constituted by PC oxygen atoms, lie just below the conduction band edge. The M1 and B1 configurations show an intermediate situation with respect to B2 and P1 configurations, where the PC molecular states lie just above the valence band edge, determining a slight decrease of the energy gap between 0.02 and 0.31 eV with respect to the pristine (101) and (001) TiO_2_ NPs. These results suggest a sizable red-shift of the optical band gap of the TiO_2_ NP upon PC adsorption, the red-shift being more consistent for the high-energy configurations, which can be supposed to be a first step in the formation of the more stable adsorbate (B2) for which, however, a deeper investigation would be needed. Remarkably, these outcomes are in agreement with those of Soria et al., who, by employing a B3LYP level of theory, investigated the TiO_2_ electronic structure modification upon adsorption of the H_2_PO_4_^–^ anion and various oligonucleotides and verified the presence of middle gap states limited to the lower in energy configurations, in contrast to the more stable in energy ones.^61^ Accordingly, although the use of a hybrid functional would reinforce the employed theoretical framework, we are confident that the PBE functional adequately describes the electronic structures in the PC-TiO_2_ adsorbates.

**Figure 4:**
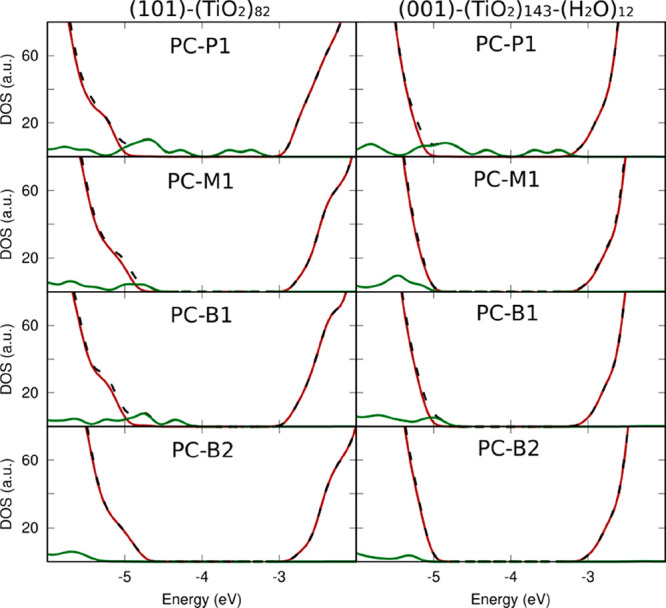
Total (DOS, black dashed line) and projected (PDOS, TiO_2_ NP in brown and PC in green solid lines, respectively) density of states for the PC adsorbates on the (101) and (001) anatase TiO_2_ NPs (see text for details).

*(101)-(TiO_2_)_82_-POPC and (001)-(TiO_2_)_143_-(H_2_O)_12_-POPC Structures*. For the more stable in energy PC adsorbates, (101)-PC-B2 and (001)-PC-B2, we considered the correspondent adsorbates involving the real POPC and two additional structures, labeled as (101)-POPC-B3 and (001)-POPC-B3, still having the same anchoring pattern of B2 structures, but with the acyl chains rotated and aligned parallel to the oxide surface, so that weak nonpolar interactions between the partners can be eventually accomplished (see Figure 5). Both the optimized (101)-POPC-B2 and (001)-POPC-B2 structures show Ti–O bond distances very similar to the correspondent PC structures (see Table 1), thus suggesting that the inclusion of the acyl chains does not significantly alter the geometries, while the adsorption energies suggest a significant stabilizing role of the acyl chains, with computed values equal to −4.08 and −3.21 eV, respectively, for the (101)-POPC-B2 and (001)-POPC-B2 structures, that is, 0.62 and 0.25 eV more stable in energy than the correspondent PC adsorbates. The POPC-B3 structures show the acyl chains aligned to the oxide surface, thus simulating an eventual disruption of the lipid double layer upon anchoring on the surface. The Ti–O bond distances show fairly the same values of the correspondent POPC-B2 structures (see Table 1), the differences being within 0.1 Å, but the effect on the adsorption energies is significant, with stabilization of 1.51 and 1.32 eV for the (101)-POPC-B3 and (001)-POPC-B3 vs the (101)-POPC-B2 and (001)-POPC-B2, respectively. The POPC-B3 configurations can be considered an evolution step of the POPC adsorption on the titanium oxide surface, toward the disruption of the lipid membrane and therefore of the death of the pathogen.

**Figure 5:**
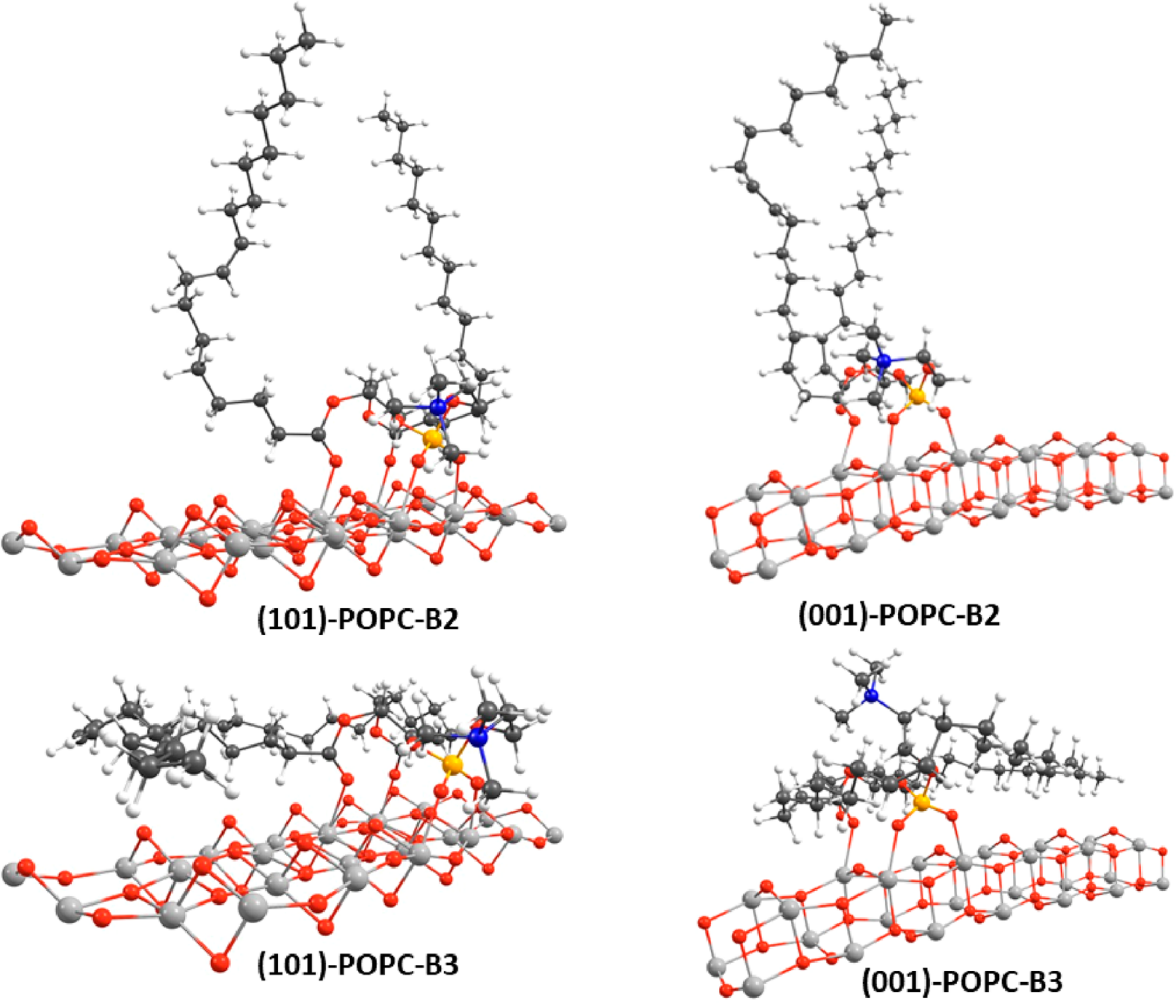
Optimized geometries for the adsorption of POPC on the (101)-(TiO_2_)_82_ (left side) and (001)-(TiO_2_)_243_-(H_2_O)_12_ (right side) clusters (only a limited surface section is shown for clarity); see text for structure labeling. Titanium atoms in light gray, oxygen in red, carbon in dark gray, phosphorus in orange, nitrogen in blue, and hydrogen in white.

As a first step toward the comprehension of the working principles of antiviral surfaces, the anchoring of the POPC molecule on the titania NP surfaces has been investigated by first-principles PBC-DFT calculations. The structures and adsorption energies of several adsorption modes of the PC model on both the (101) and (001) surfaces of TiO_2_ NPs have been analyzed, finding 4 different configurations stable in energy with respect to the reactants. In particular, one structure describes a physisorption of the PC molecule on the TiO_2_ surface (P1), with a sizable stabilization energy of −0.91 and −0.72 eV on the (101) and (001) surfaces, respectively. The remaining 3 structures are characterized by stronger adsorption energies, in the ranges of −1.7 and −3.5 eV, because of the formation of covalent bonds between the semiconductor Ti_5c_ sites and the PC nucleophilic sites. Remarkably, the (101) and (001) surfaces show a different order in the energy stability of the monodentate (M1) and bidentate (B1) configurations, where one and two covalent bonds are engaged, respectively, between the Ti_5c_ sites and the PC phosphate oxygens. On the (101) surface, the M1 configuration is 0.39 eV more stable than B1, while on the (001) surface, the M1 configuration is 0.50 eV higher than B1. For both the (101) and (001) surfaces, the bidentate B2 configuration is computed to be the most stable in energy, with values of −3.46 and −3.21 eV, respectively. The optimized geometries show that the (101)-B2/(001)-B2 structures engage two Ti_5c_–O–P covalent bonds, as in B1, and in addition two/one Ti–O bonds involving the ester carbonyl oxygen atoms on the PC residue. Analysis of the electronic structure of the optimized structures shows a correlation between the orbital energy gap of adsorbed POPC on TiO_2_ and their interaction energy. The most stable adsorption configuration (B2) shows a clean energy gap with the PC molecular states lying within the valence and conduction bands of the TiO_2_ NP. A weaker interaction of PC on the oxide surface favors the energy increase of the PC occupied molecular states to the valence band edge, introducing occupied states in the semiconductor energy gap, while the empty states, lying higher in energy, are not perturbed.

Our results point out that the stability of the adsorbate and the strength of the attachment are strictly related to the peculiar nature of POPC, with marked differences with respect to simple phosphate species. The presence of the acyl chains strongly contributes to the stabilization in energy of the adsorbates and the B3 configuration, where the acyl chains are rotated by about 90° with respect to the orientation in the double layer lipidic membrane, may be considered a step toward the disruption of the membrane protecting the virus and therefore toward the death of the enveloped virus.

## Supporting Information Available

Main bond distances, adsorption energies, and discussion of the PC adsorbates on the (101)-(TiO_2_)_38_ NP; main bond distances and adsorption energies for the PC adsorbates on the (101)-(TiO_2_)_82_ and (001)-(TiO_2_)_143_-(H_2_O)_12_ NPs computed without the inclusion of the dispersion forces; optimized geometries for the PC adsorbates on the (101)-(TiO_2_)_38_ NP (PDF)
