# Supplementary material for: Modeling the Interaction of Coronavirus Membrane Phospholipids with Photocatalitically Active Titanium Dioxide
Source: J Phys Chem Lett. 2023 Jun 21;14(25):5914–23. doi: 10.1021/acs.jpclett.3c01372 (PMC10316400; doi:10.1021/acs.jpclett.3c01372)
Supplement: Supplementary file 1 — jz3c01372_si_001.pdf [file jz3c01372_si_001.pdf]

Modeling The Interaction Of Coronaviruses Membrane Phospholipds With Photocatalitically Active Titanium Dioxide

Iván Soriano-Díaz,[1,6] Eros Radicchi,[2] Beatrice Bizzarri,[1,3] Olivia Bizzarri,[1] Edoardo Mosconi,[3] Muhammad W. Ashraf,[4] Filippo De Angelis,[1, 3, 4, 5*] Francesca Nunzi,[1,3*]

[1] Department of Chemistry, Biology and Biotechnology, University of Perugia, Via Elce di Sotto 8, 06123 Perugia, Italy.

[2] Nanomaterials Research Group, Department of Biotechnology, University of Verona, Strada Le Grazie 15, Verona, Italy.

[3] Computational Laboratory for Hybrid/Organic Photovoltaics (CLHYO), Consiglio Nazionale delle Ricerche (CNR) - Istituto di Scienze e Tecnologie Chimiche "Giulio Natta" - SCITEC, Via Elce di Sotto 8, 06123 Perugia, Italy.

[4] Department of Natural Sciences and Mathematics, College of Sciences and Human Studies, Prince Mohammad Bin Fahd University, Khobar, Dhahran 34754 Saudi Arabia.

[5] SKKU Institute of Energy Science and Technology (SIEST), Sungkyunkwan University, Suwon 440-746, Korea.

[6] Instituto de Ciencia Molecular, Universidad de Valencia, 46980 Paterna, Spain

Supporting Information

(101)-$(TiO_2)_{38}$-PC structures. Preliminary calculations at a lighter computational cost have been carried out by employing the reduced models for both the partners, i.e. the (101)-$(TiO_2)_{38}$ cluster model and the PC molecule. On overall, we gained four adsorption structures stable in energy with respect to the isolated partners (see Figure S2 and Table S1 in the SI section). The structure associated to the physisorption process on the $(TiO_2)_{38}$ NP surface, labelled as P1, has an adsorption energy equal to -0.92 eV, probably due to dispersion interactions between the diglycerol ester residue and the titanium surface, while the positive choline residue is oriented away from the surface. Among the chemisorbed structures, M1 shows a monodentate Ti-O-P bonding mode with respect to the phosphate group, but has an additional Ti-O-C bond with one ester carbonyl oxygen. The two covalent bonds give rise to an adsorption energy of even -2.34 eV. In addition, two bridging structures, B1 and B2, have been optimized, where the two phosphate oxygens are bonded to undercoordinated Ti sites. In the B2 structure a third bond is engaged, involving one ester carbonyl oxygen, thus leading to the most stable in energy structure for the $(TiO_2)_{38}$-PC adsorbates, with an adsorption energy of -2.82 eV. The B1 structure shows an absorption energy of only 0.98 eV, probably due to a high energy strain related to a high distortion of the reduced TiO_2 cluster model upon PC attachment. The optimization of the $(TiO_2)_{38}$-PC structures allows us to acquire a sensibility on the anchoring possibilities of POPC on the titanium dioxide surface, but the $(TiO_2)_{38}$ cluster model is too small to permit an adequate description of the POPC interaction on the surface.

(101)-$(TiO_2)_{38}$				
	**PC-P1**	**PC-M1**	**PC-B1**	**PC-B2**
**Ti-O_{1P}**	4.50 (4.51)	2.11 (2.12)	2.08 (2.09)	2.17 (2.21)
**Ti-O_{2P}**	5.23 (5.18)	2.66 (2.76)	2.19 (2.24)	2.09 (2.08)
**Ti-O_{3C}**	5.51 (5.59)	2.98 (3.00)	7.18 (7.95)	2.33 (2.35)
**Ti-O_{4C}**	2.94 (2.98)	2.14 (2.16)	4.90 (4.91)	2.80 (2.81)
**E_{ads}**	-0.92 (0.01)	-2.34 (-0.77)	-0.98 (0.13)	-2.82 (-1.51)

Table S1. Main bond distances (in Angstrom), adsorption energies (E_{ads}, in electronvolt) for the PC adsorbates on the (101)-$(TiO_2)_{38}$ NP. In parenthesis the value computed without the inclusion of the dispersion forces are reported.

(101)-$(TiO_2)_{82}$						
	**PC-P1**	**PC-M1**	**PC-B1**	**PC-B2**	**POPC-B2**	**POPC-B3**
**Ti-O_{1P}**	3.68	2.21	2.02	2.21	2.19	2.16
**Ti-O_{2P}**	5.14	2.23	3.94	2.18	2.16	2.18
**Ti-O_{3C}**	6.05	4.11	5.26	2.38	2.38	2.39
**Ti-O_{4C}**	3.47	5.02	3.56	2.38	2.27	2.27
**E_{ads}**	0.01	-0.86	-0.94	-1.74	-2.56	-2.75
**(001)-$(TiO_2)_{143}$-$(H_2O)_{12}$**						
	**PC-P1**	**PC-M1**	**PC-B1**	**PC-B2**	**POPC-B2**	**POPC-B3**
**Ti-O_{1P}**	4.84	2.11	2.14	2.12	2.12	2.05
**Ti-O_{2P}**	5.58	3.38	2.25	2.13	2.13	2.26
**Ti-O_{3C}**	5.75	5.41	3.75	2.37	2.34	2.34
**Ti-O_{4C}**	2.71	4.46	3.10	2.97	3.04	3.40
**E_{ads}**	-0.08	-0.78	0.76	-1.55	-2.27	-1.72

Table S2. Main bond distances (in Angstrom), and adsorption energies (E_{ads}, in electronvolt) for the PC/POPC adsorbates on the (101)-$(TiO_2)_{82}$ (top) and (001)-$(TiO_2)_{143}$-$(H_2O)_{12}$ (bottom) NPs computed without the inclusion of the dispersion forces.

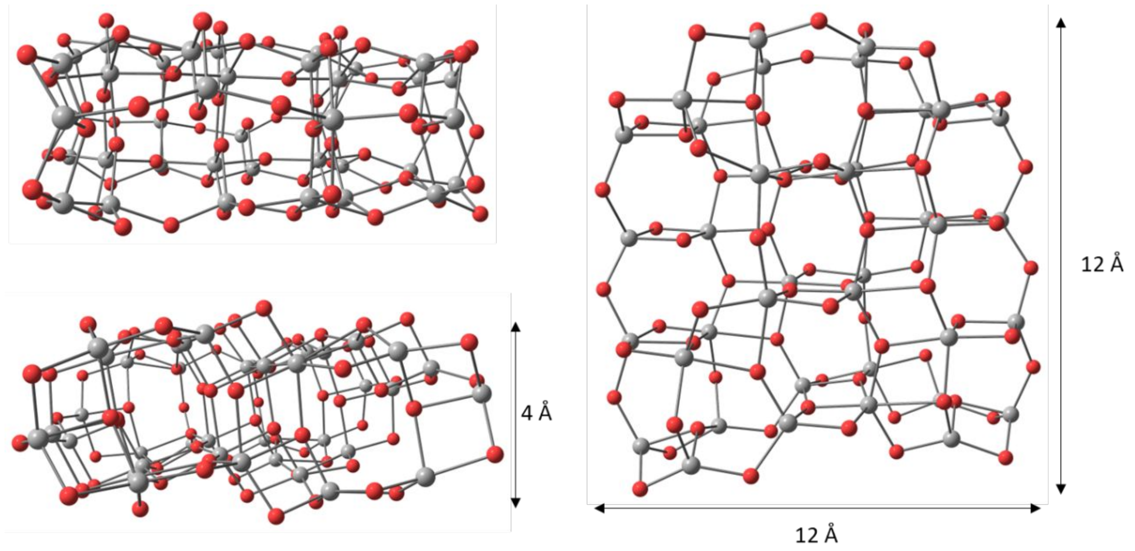

Figure S1. Optimized geometries (top and side views) for the $(TiO_2)_{38}$ cluster model, exposing the (101) surface. Titanium atoms in light grey, oxygen in red.

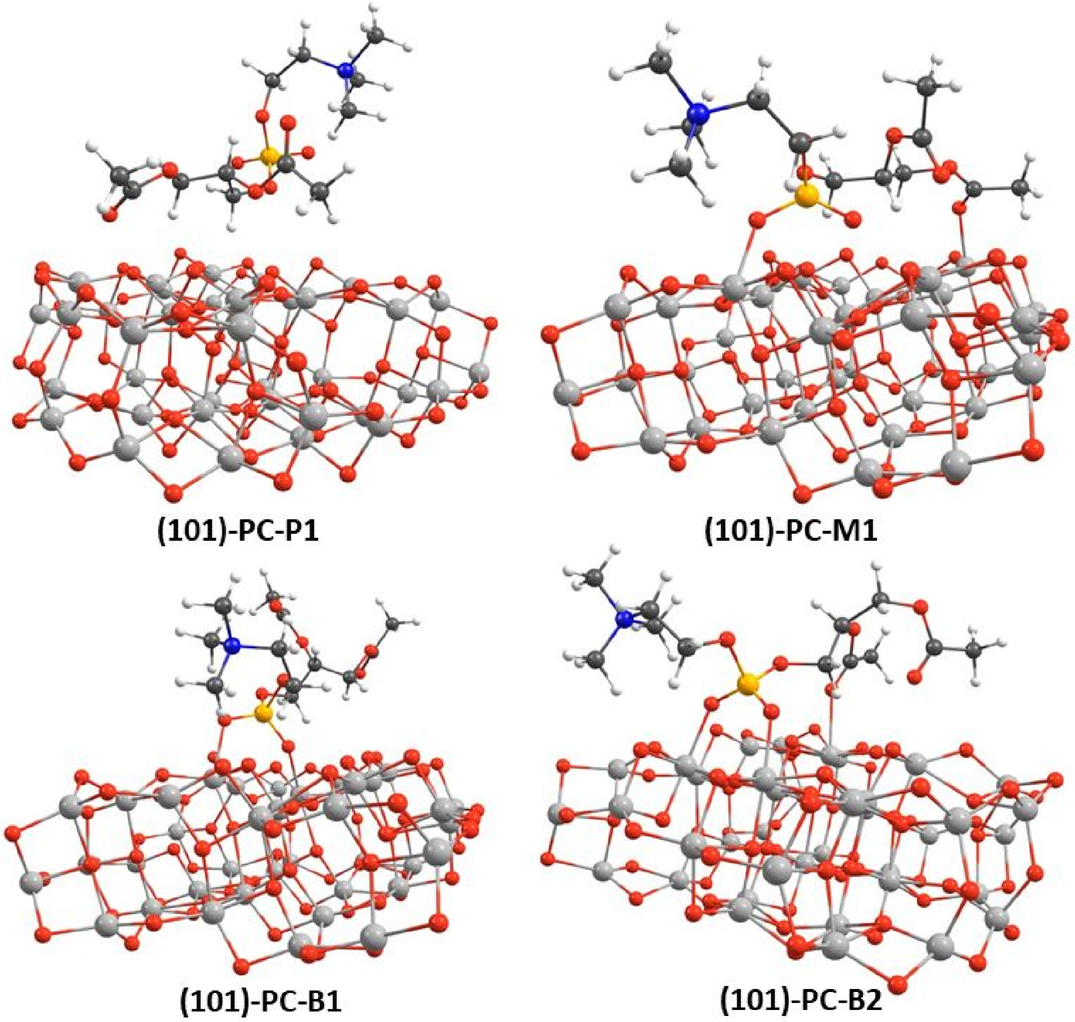

Figure S2. Optimized geometries for the adsorption of PC on the (101)-$(TiO_2)_{38}$ cluster, see text for structure labelling. Titanium atoms in light grey, oxygen in red, carbon in dark grey, phosphorus in orange, nitrogen in blue and hydrogen in white.

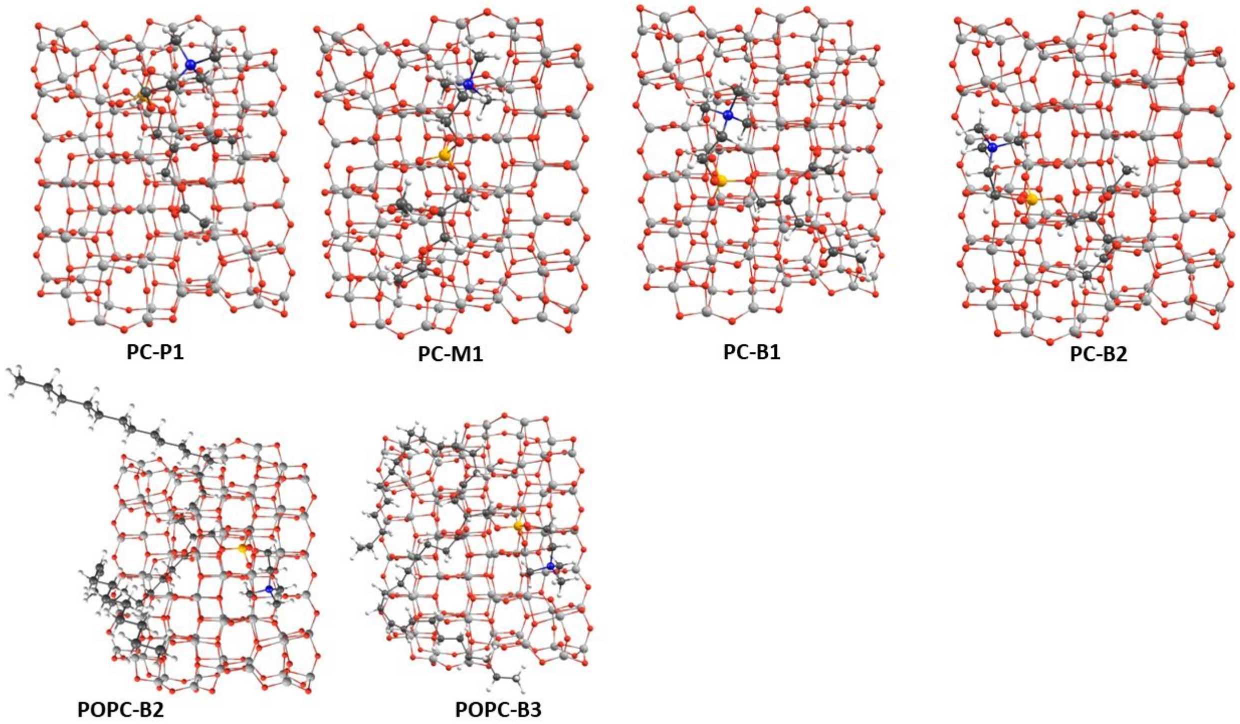

Figure S3. Optimized geometries for the adsorption of PC on the (101)-$(TiO_2)_{82}$ cluster, see text for structure labelling. Titanium atoms in light grey, oxygen in red, carbon in dark grey, phosphorus in orange, nitrogen in blue and hydrogen in white.

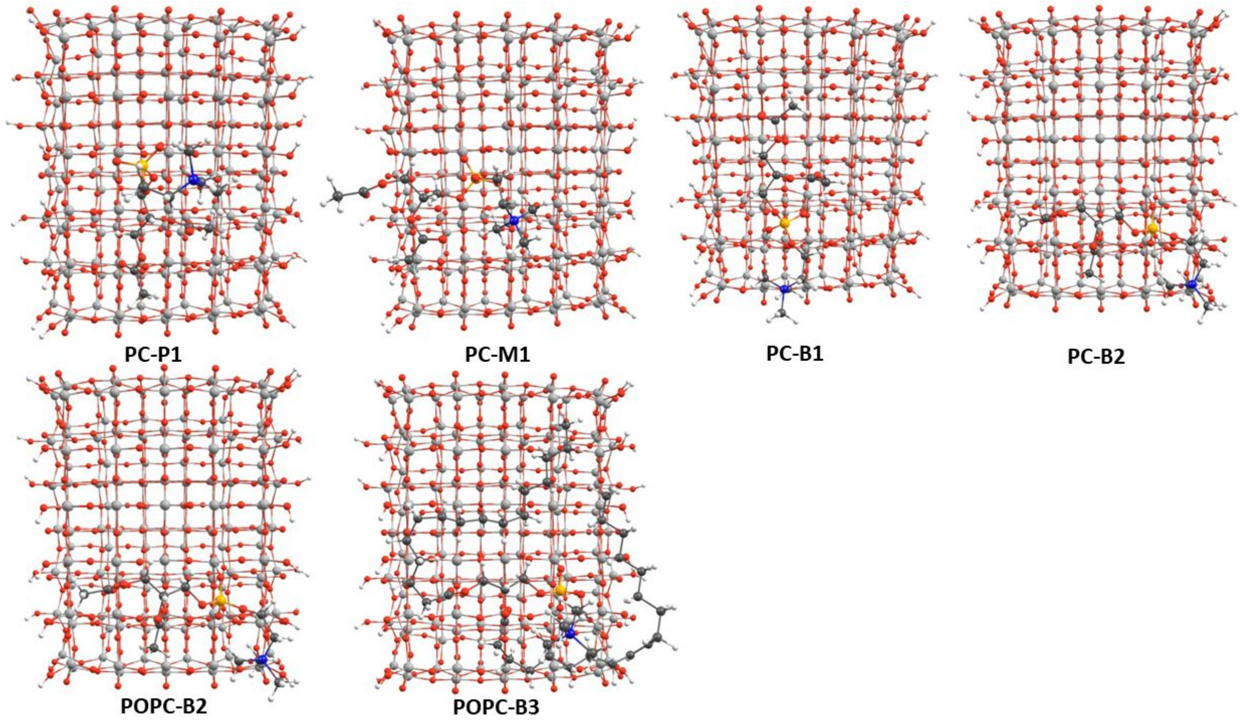

Figure S4. Optimized geometries for the adsorption of PC on the (100)-$(TiO_2)_{243}$-$(H_2O)_{12}$, see text for structure labelling. Titanium atoms in light grey, oxygen in red, carbon in dark grey, phosphorus in orange, nitrogen in blue and hydrogen in white.
